# Identifying Malaria Transmission Foci for Elimination Using Human Mobility Data

**DOI:** 10.1371/journal.pcbi.1004846

**Published:** 2016-04-04

**Authors:** Nick W. Ruktanonchai, Patrick DeLeenheer, Andrew J. Tatem, Victor A. Alegana, T. Trevor Caughlin, Elisabeth zu Erbach-Schoenberg, Christopher Lourenço, Corrine W. Ruktanonchai, David L. Smith

**Affiliations:** 1 WorldPop Project, Geography and Environment, University of Southampton, Southampton, United Kingdom; 2 Flowminder Foundation, Stockholm, Sweden; 3 Department of Mathematics, Oregon State University, Corvallis, Oregon, United States of America; 4 School of Forest Resources and Conservation, Gainesville, Florida, United States of America; 5 Clinton Health Access Initiative, Boston, Massachusetts, United States of America; 6 Institute for Health Metrics and Evaluation, University of Washington, Seattle, Washington, United States of America; 7 Fogarty International Center, National Institutes of Health, Bethesda, Maryland, United States of America; 8 Sanaria Institute for Global Health and Tropical Medicine, Rockville, Maryland, United States of America; 9 Spatial Epidemiology and Evolution Group, Department of Zoology, University of Oxford, Oxford, United Kingdom; Duke University, UNITED STATES

## Abstract

Humans move frequently and tend to carry parasites among areas with endemic malaria and into areas where local transmission is unsustainable. Human-mediated parasite mobility can thus sustain parasite populations in areas where they would otherwise be absent. Data describing human mobility and malaria epidemiology can help classify landscapes into parasite demographic sources and sinks, ecological concepts that have parallels in malaria control discussions of transmission foci. By linking transmission to parasite flow, it is possible to stratify landscapes for malaria control and elimination, as sources are disproportionately important to the regional persistence of malaria parasites. Here, we identify putative malaria sources and sinks for pre-elimination Namibia using malaria parasite rate (PR) maps and call data records from mobile phones, using a steady-state analysis of a malaria transmission model to infer where infections most likely occurred. We also examined how the landscape of transmission and burden changed from the pre-elimination setting by comparing the location and extent of predicted pre-elimination transmission foci with modeled incidence for 2009. This comparison suggests that while transmission was spatially focal pre-elimination, the spatial distribution of cases changed as burden declined. The changing spatial distribution of burden could be due to importation, with cases focused around importation hotspots, or due to heterogeneous application of elimination effort. While this framework is an important step towards understanding progressive changes in malaria distribution and the role of subnational transmission dynamics in a policy-relevant way, future work should account for international parasite movement, utilize real time surveillance data, and relax the steady state assumption required by the presented model.

## Author Summary

For countries considering pursuing malaria elimination, understanding where malaria transmission occurs is crucial for intervention planning. By identifying the areas that act as sources of malaria parasites, elimination programs can target efforts to end local transmission and achieve nationwide elimination. Mapping parasite sources requires a modeling framework that integrates malaria burden and human movement information, however, as human mobility facilitates parasite spread and drives source-sink disease dynamics. In this study, we present a mathematical model that can be used to identify areas with self-sustaining malaria transmission when analyzed at equilibrium. We demonstrate how this method can inform elimination planning for countries with stable low transmission using data from Namibia. The maps of sources and sinks created using this method can be used to direct policy and target areas with self-sustaining malaria transmission in countries with stable transmission. Finally, we compare the predicted extent of transmission foci with more recent maps of incidence, to determine whether local transmission likely retreated into focal areas and the potential importance of importation.

## Introduction

Human malaria is caused by infection with *Plasmodium falciparum* and four other species of parasites, accounting for around 600,000 deaths and 100–250M febrile episodes annually [1]. The global burden of malaria is declining, partly due to an increase in donor funding and scaled up distribution of vector control and effective medicines [1,2]. Many countries have set the goal of eliminating malaria in the coming decades, which involves stopping transmission, emptying the parasite reservoir, and then managing imported malaria and potential outbreaks [3]. To continue this progress, methods of prioritizing particular areas for targeting control efforts have been proposed [4,5], requiring an understanding of the spatial patterns of transmission.

Since mosquitoes transmit the pathogen, and since the underlying distributions of mosquitoes and humans are highly heterogeneous [6], so is the intensity of transmission [4,7]. Heterogeneity in transmission is observed throughout on the road to elimination, providing an opportunity for spatial targeting of control [4,7]. This heterogeneity in malaria transmission is driven by ecological and social factors such as Anopheline mosquito density, land use and agricultural practices, bed net use, wealth and education, access to and utilization of healthcare, and urbanization [8,9], and these factors drive heterogeneity across all spatial scales, from very local [5] to national and global landscapes [10]. Because of these spatially heterogeneous processes that drive transmission, malaria tends to persist in “foci”, or localized areas of self-sustaining transmission [5]. Malaria burden can also be relatively high outside of foci, as human and mosquitoes transport parasites [4,11]. Because they are the ultimate sources of local parasites, foci drive the spatial distribution of endemic malaria, and effective targeting requires their identification and understanding how human populations interact with them [12].

Some statistical algorithms and *ad hoc* methods target foci for control [5,13] and quantify change in their spatial extents [13]. Despite this, no formal mathematical or mechanistic definition of a malaria focus exists, limiting practical application and ability to explain and predict changes in focal extent over time. One possible quantitative definition regards it as a demographic source or an area where reproductive success is, on average, high enough for some population to persist and export individuals [14]. This definition is well-established, as spatial heterogeneity in demographic success and population dynamics has been explored in detail in ecological literature [14,15]. In this context, sources are self-sustaining areas of high demographic success, or areas where birth rates exceed death rates and excess individuals are exported [14]. Sinks, on the other hand, are areas where populations require a constant flow of immigrants to persist [14].

For malaria parasites and other pathogens, demographic success is defined by reproductive numbers [16]. In this case, a source is where an infection in one host tends to be propagated to more than one host and excess parasites tend to be exported to other areas [11,17,18]. For malaria, the basic reproductive number *R*_*0*_ has been both a useful threshold criterion for endemicity and a basis for setting intervention coverage targets. If *R*_*0*_ > 1, malaria is expected to persist locally because each case causes more than one case, whereas pathogen transmission is not sustained over time if *R*_*0*_ < 1[16].

**Fig 1:**
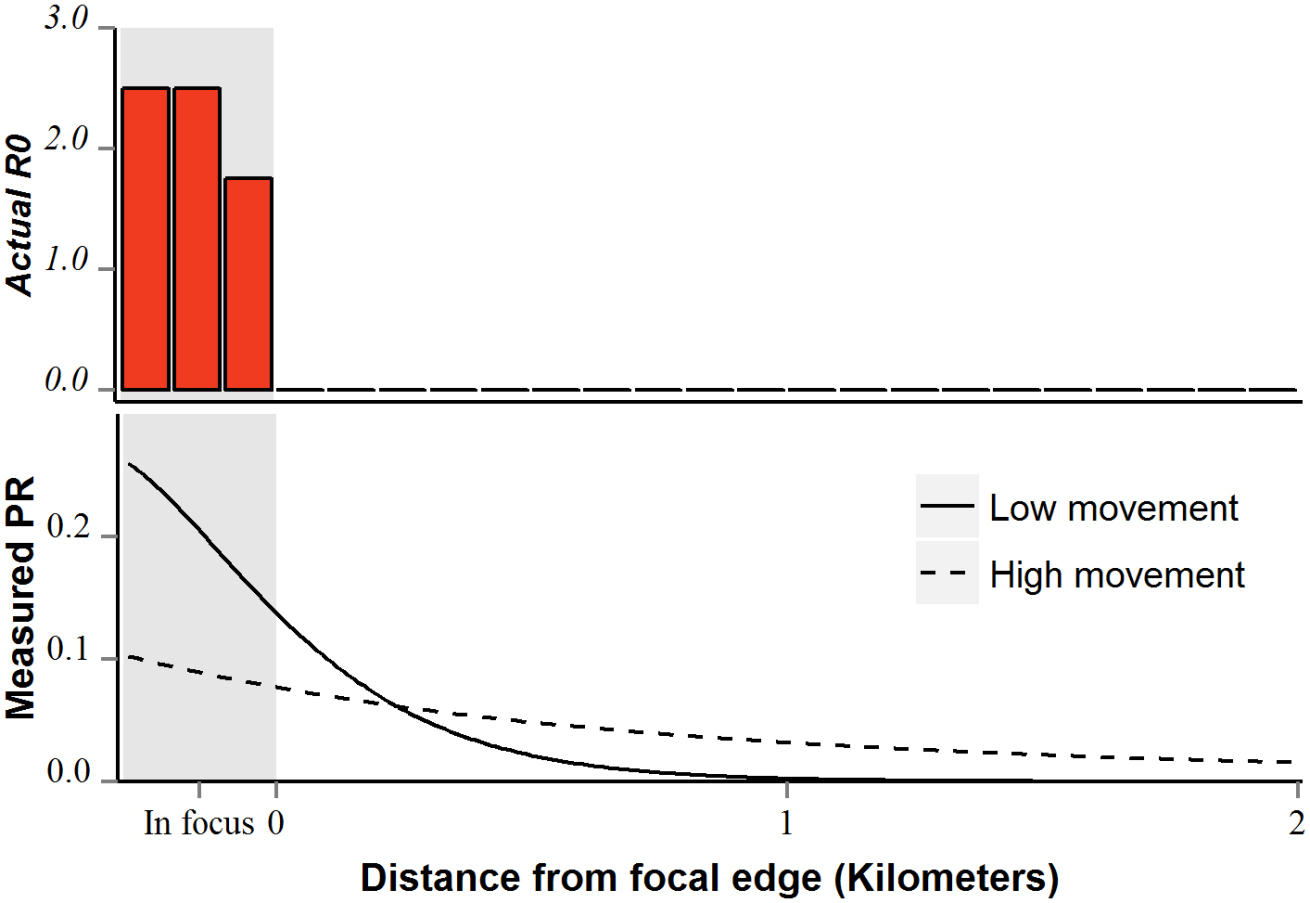
Hypothetical observed fraction of people infected with malaria (parasite rate) within and near malaria foci. Transmission only occurs within focal areas (top panel). Observed parasite rate shown in bottom panel with varying distances travelled. As humans move further and/or more frequently from within focal areas, they export parasites, causing larger extra-focal extents to exhibit nonzero parasite rate (dotted line; bottom panel).

The conventional definition of *R*_*0*_ describes transmission in some particular place disregarding malaria importation and the spatial configuration of malaria transmission [19]. In fact, malaria often persists in areas where *R*_*0*_ < 1, however, due to human and mosquito movement [20], termed “non-endemic transmission” [21,22]. This non-endemic transmission is driven by both importation via incoming migrants [23], and by residents infected with parasites during travel [23,24]. As humans move more frequently and further, more areas outside of foci receive parasites via mosquito and human movement. These extra-focal areas may then harbor a nonzero fraction of people infected with malaria, or a nonzero parasite rate (Fig 1). Extra-focal areas with infected people are ultimately demographic sinks of parasites, as parasite death rates exceed birth rates locally (represented by a local *R*_*0*_ < 1) but those parasite populations are sustained through immigration [14].

**Fig 2:**
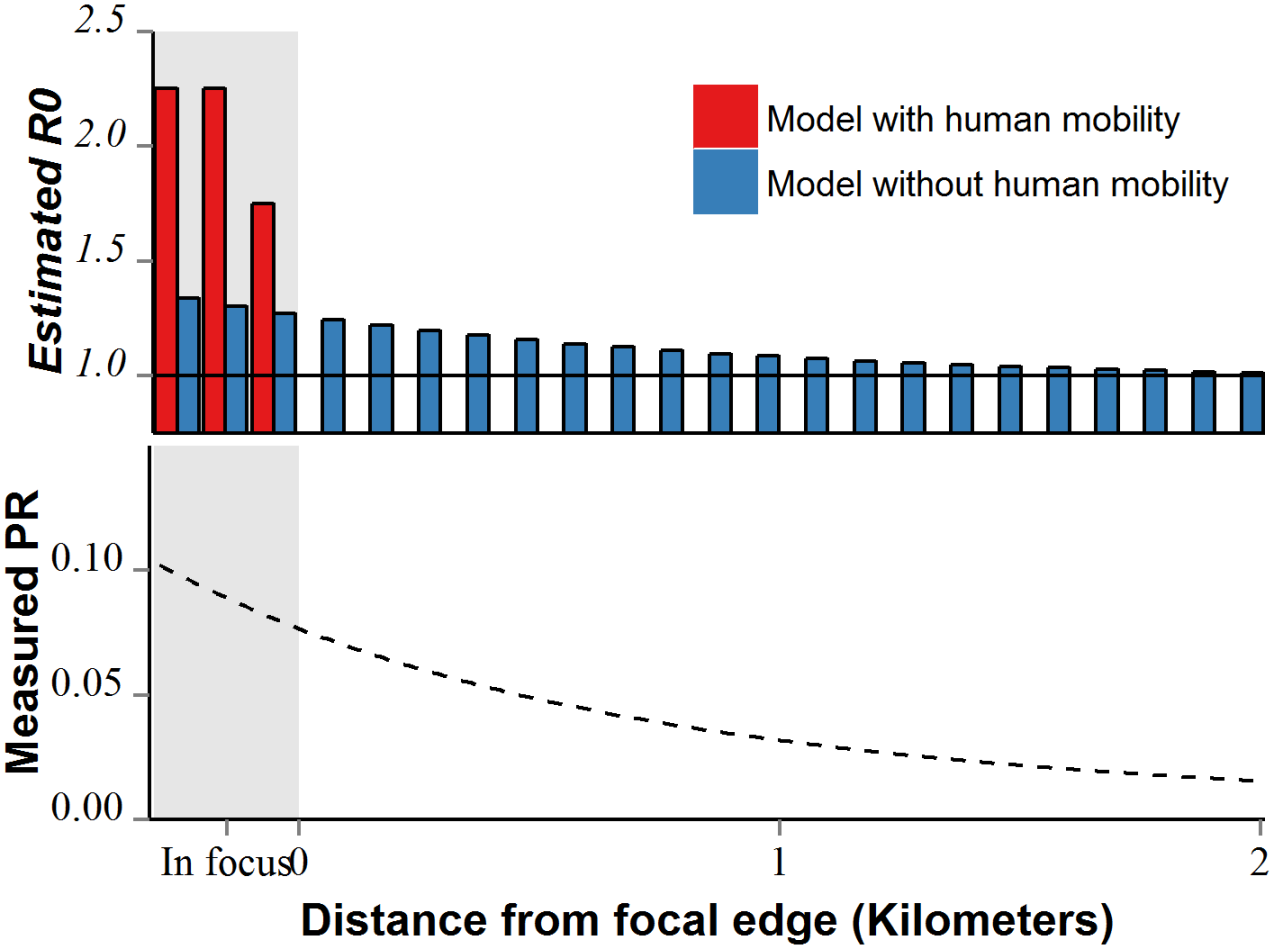
Estimating local transmission intensity using a patch-based model with human mobility and a classical model without mobility. In reality, transmission only occurs inside a transmission focus, though extra-focal patches may exhibit nonzero parasite rate at equilibrium due human-mediated parasite movement (bottom panel). Using a classical model without human mobility to estimate transmission capacity from PR (assuming PR is static) leads to the incorrect conclusion that all patches with nonzero PR have a basic reproductive number above 1 (top panel; blue). By accounting for human movement, a patch-based model can accurately determine the patches where transmission occurs (top panel, red).

If burden is assumed to be stable spatially and temporally, ignoring parasite mobility leads to the incorrect conclusion that all malaria-endemic areas would have self-sustaining parasite populations [25]. Micro-epidemiological [26] and malaria metapopulation models [20] can be used to identify which of these areas are actually transmission foci (*i*.*e*. sources, when paired with extra-focal human movement) by quantifying migration and modelling transmission within and among populations. By applying these methods to define transmission foci, novel methods for spatial targeting of control can accurately quantify *R*_*0*_ and identify areas with sustainable transmission (Fig 2). These methods can then improve efficiency in achieving important policy goals such as regional malaria elimination by guiding control targeting efforts.

Here, we demonstrate use of a method for identifying putative sources and sinks of malaria transmission in a multipatch setting at steady state with respect to prevalence (or parasite rate). Using malaria prevalence estimates with a call record dataset from mobile phones, we calculate the transmission patterns necessary to yield observed patterns of prevalence, assuming burden was not changing over time. We discuss this method’s utility for informing future elimination efforts in countries where malaria burden is generally low but stable, as transitioning towards elimination in these settings means concentrating activities on identifying and attacking transmission foci [3]. As we analyze the presented model at equilibrium, it is not appropriate in areas where burden is changing through time.

In this case study, we use prevalence estimates from the pre-elimination phase in Namibia [10] with 2010 mobile phone call record data to identify pre-elimination transmission foci. Because the survey data informing the prevalence estimates for Namibia used originated largely from pre-2000 [10], we assume these estimates represent a period when burden was relatively stable (matching the steady state assumption required by our method). Overall control was relatively limited during this time as Global Fund-supported efforts began in the early 2000s [27], and case numbers did not drop significantly until after 2004 [1,27]. Stability of burden cannot be confirmed with certainty without more historical data, however, as national-level incidence data were not available from before 2001 [1]. As the output maps show historical transmission foci, practical application of the presented results is limited, though these historical foci allow us to compare against more recent malaria burden estimates to examine possible change in the spatial patterns of transmission over time. We compare the predicted transmission foci map with an incidence map reflecting burden in 2009 [26] to assess how transmission patterns may have changed between these time periods. We also use this analysis to assess the possible importance of internationally imported cases into non-focal areas on overall case distribution.

## Results

We employed a multipatch version of the Ross-Macdonald malaria transmission model, modifying a previously published model [20] to integrate human movement in a way that could be parameterized using mobile phone records. Local data were used to calculate the “partial” local vectorial capacity, denoted *C*_*i*_. The partial vectorial capacities were used to calculate a patch-specific “local reproductive number” *R*_*0*,*i*_ using eq 5 (see Methods) to determine whether an area is a source / focus. This local reproductive number represents the number of new cases expected from a single infected individual in a completely naïve population, if the patch is completely isolated from the system.

### National-level case study

To demonstrate how this modeling framework can guide elimination planning, we used our model to identify transmission foci and quantify parasite flow throughout Namibia using parasite rate estimates. We compared the results of steady state analyses of this multipatch model with similar analysis of a classical model without human mobility. Table 1 contains the values of various malaria metric parameters used for both models, and are constant throughout all analyses. The parameters used originate from African vectors in The Gambia [28,29], laboratory trials with *Anopheles gambiae* mosquitoes[30,31], the most common malaria vector in Africa, and epidemiological studies [32,33]. Figures do not show areas initially estimated to have zero parasite rate, as neither model predicted any areas with zero PR to have *R*_*0*,*i*_ > 1 when analyzed at equilibrium. Further, while the multipatch model provides an estimate of *R*_*0*,*i*_ for patches where parasite rate is zero at equilibrium, the classical model without movement can only estimate that *R*_*0*,*i*_ < 1 without providing an exact estimate [25].

**Table 1:** Model parameter definitions and values of each parameter used in process validation simulations.

Parameter	Biological Interpretation	Value used in model
*a*	Number of bites on humans per day, per mosquito	0.3[28,29]
*b*	Transmission efficiency from mosquito to human	0.10[28]
*c*	Transmission efficiency from human to mosquito	0.214[30,31]
*r*	Human recovery rate, in days	1/150[32,33]
*μ*	Mosquito death rate, per day	0.1[28]

#### Parasite rate estimates

**Fig 3:**
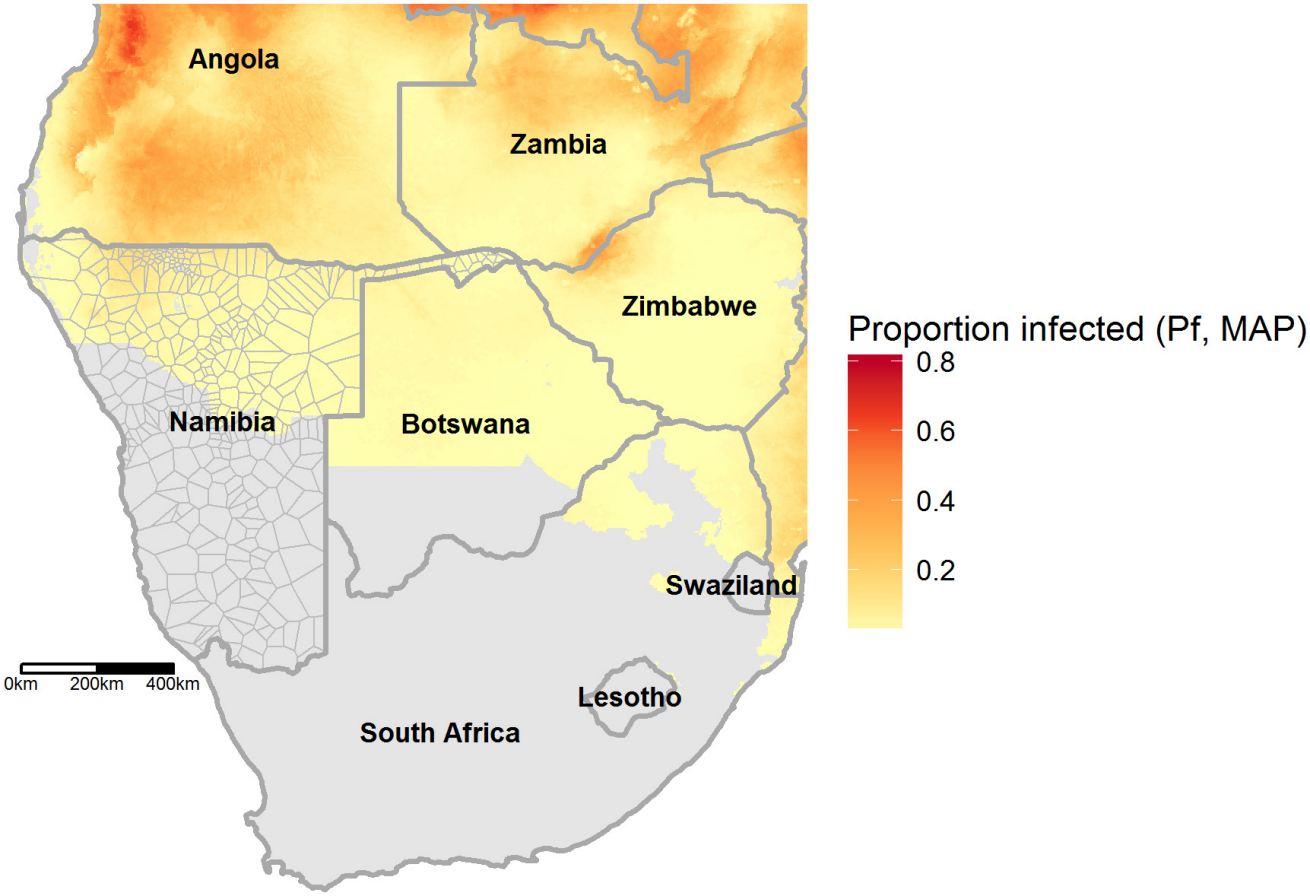
Regional map with parasite rate estimates from [10]. *Plasmodium falciparum* parasite rate (*Pf*PR) shown in yellow and red; grey areas indicate areas with no malaria or areas where predicted annual incidence is less than 1 per thousand people (*Pf*PR surface adapted from [10]). National borders obtained from thematicmapping.org, and are available for use under a Creative Commons BY-SA license.

**Fig 4:**
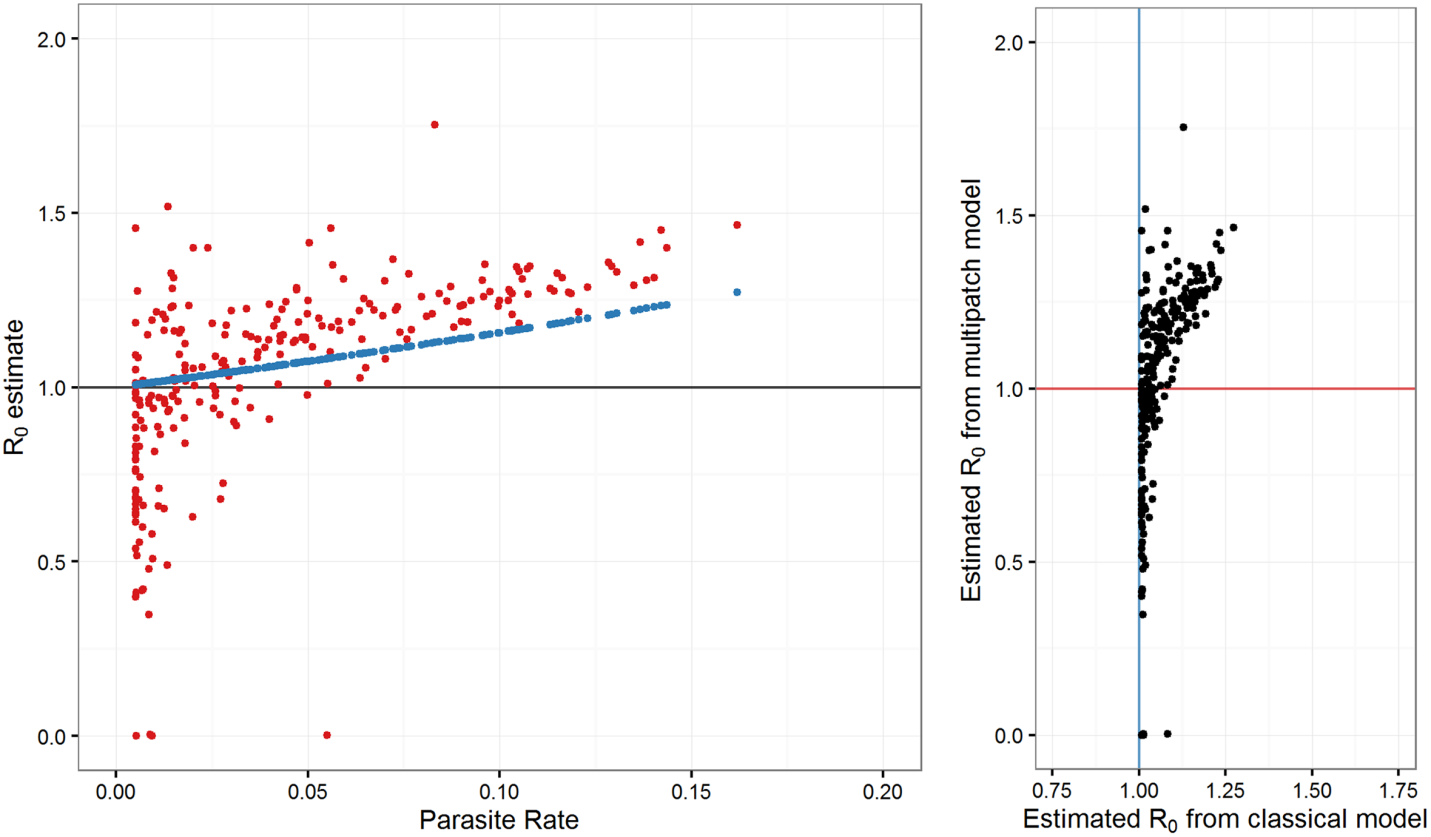
R_0_ estimates using steady state analyses of multipatch model with mobility and classical model without mobility for all patches with nonzero PR. Left panel: R_0_ estimates using both models compared with observed PR in each patch. Right panel: Relationship between R_0_ estimates from both models.

**Fig 5:**
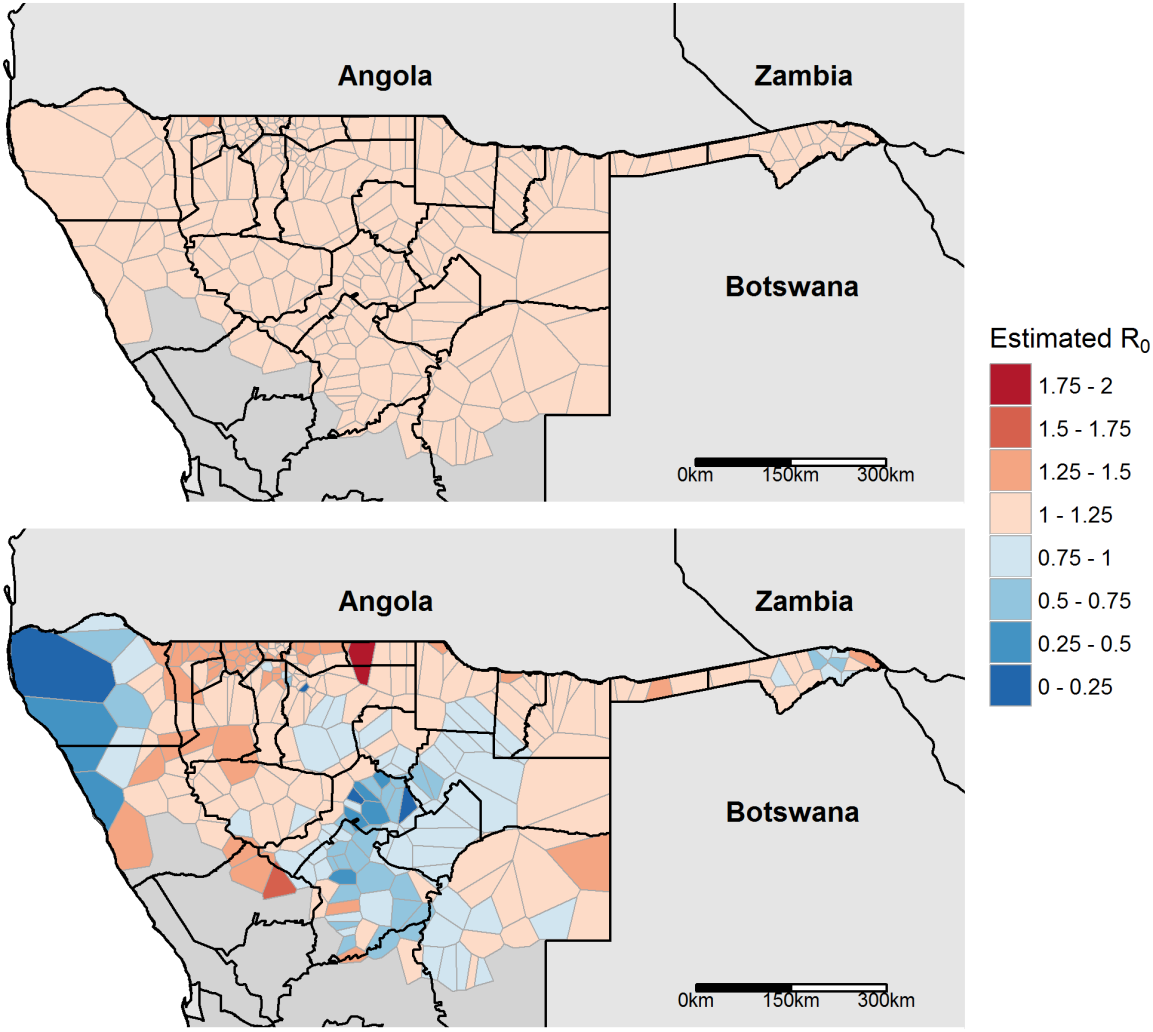
Predicted extent of transmission foci using pre-elimination parasite rate estimates. Top panel: Predictions from classical model analysed at steady state. Bottom panel: Predictions from multipatch model. Areas where R_0_ > 1 shown in shades of red while areas where R_0_ < 1 shown in shades of blue. Only areas with nonzero PR are shown; these areas were predicted to have R_0_ < 1 in both models. Patch outlines shown in grey, health district boundaries in black. In the steady state analysis of the classical model, all patches with PR > 0 are predicted to have 1.25 > R_0_ > 1 except one bordering Angola where R_0_ = 1.27 (darker red).

Fig 3 shows the parasite rate surface in Namibia and surrounding countries, obtained from the Malaria Atlas Project [10]. Fig 4 compares the resultant estimates of *R*_*0*_ using the two models, assuming the system is at equilibrium. Fig 5 plots areas throughout Namibia with their corresponding *R*_*0*_ estimates, comparing *R*_*0*_ estimates obtained from steady-state analyses of the two models in their geographical context.

**Fig 6:**
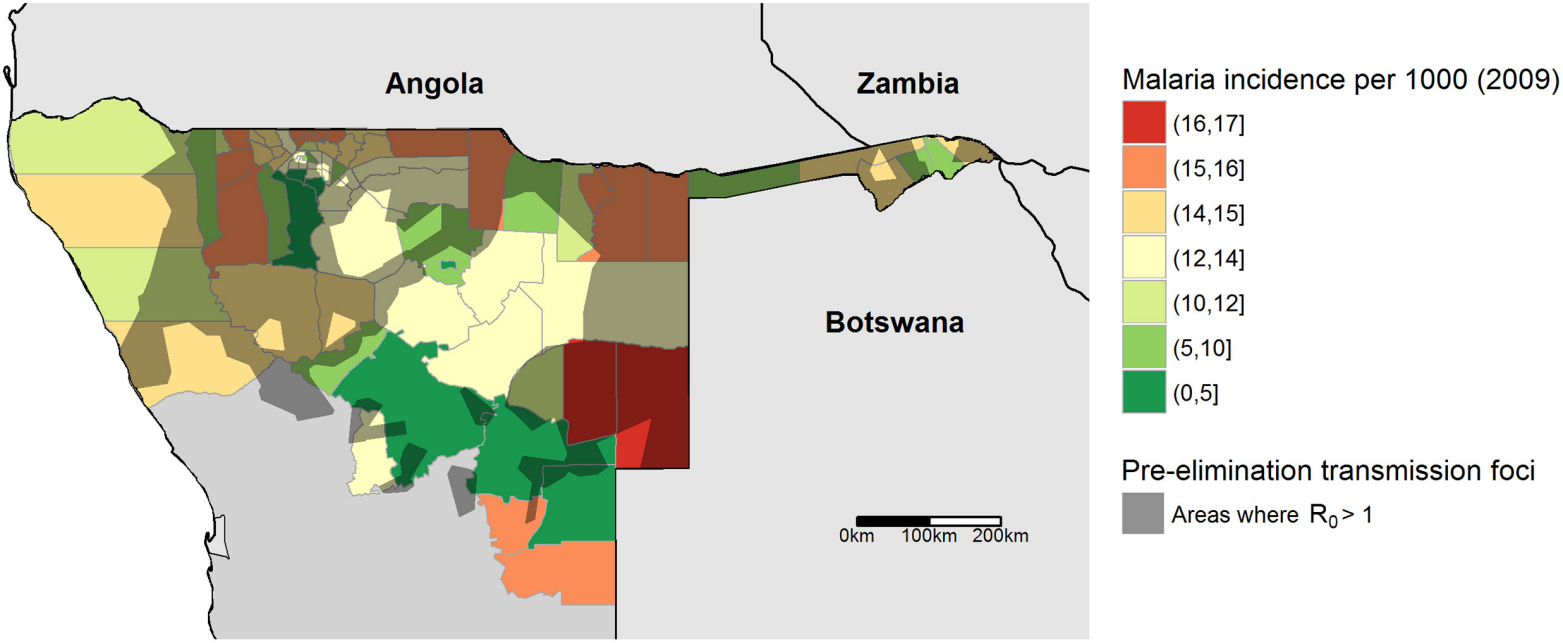
Predicted pre-elimination transmission foci overlaid on incidence for 2009. Health districts are shown, with colour corresponding to the number of predicted cases per 1000 individuals for 2009 [26]. Darkened areas represent Voronoi polygons around cell towers predicted to have *R*_*0*_ *> 1*.

The prevalence estimates used in this study were informed using prevalence surveys from before 2000 [10], representing a picture of nationwide transmission before major elimination efforts began in 2004 [34]. Understanding how case distribution patterns have changed since this period remains an important question, however, and comparing pre-elimination focal extent with later incidence estimates can help visualize changes in transmission patterns and the possible role of importation. Therefore, we compare the map of transmission foci using the MAP prevalence surface with a map of modeled incidence for 2009 [26] (Fig 6). We compared incidence within and outside of health districts, by rasterizing the transmission foci and incidence layers and comparing incidence inside and outside foci. Considering only health districts with a nonzero PR, districts overlapping transmission foci had a mean incidence of 12.5 while districts not overlapping foci had a mean incidence of 8.7.

## Discussion

Human movement is a key driver of spatiotemporal vector-borne disease dynamics, as pathogen exportation out of malaria foci sustains transmission across much larger spatial extents [21,22]. People move often and for a variety of reasons [35], including local routines [36], short-term labor-related movement, and long-term migration, and in moving they disperse parasites typically further than mosquitoes [11]. Targeting the areas responsible for malaria persistence across landscapes, then, requires a quantitative framework that integrates human movement information and disease burden data [4,37].

The presented method identifies transmission foci in settings with temporally static and generally low levels of malaria burden, using human mobility in a modeling framework rooted in ecological literature. Generally, the results produced by this study can help inform targeting efforts when elimination efforts begin in countries where burden is unchanging over time. We demonstrate use of this method in a case study with pre-elimination prevalence estimates and mobile phone data from Namibia, generating historical estimates of transmission capacity and highly resolved maps of malaria dynamics. Importantly, vectorial capacity and basic reproductive number estimates generated using this method are in the context of the transmission setting represented by the prevalence data. If a country is undergoing active malaria control, then the basic reproductive number calculated represents *R*_*c*_, or the basic reproductive number given the current level of control [38]. This method then can provide guidelines for interventions in addition to the baseline represented by the input data.

In this case study, we used prevalence estimates informed largely by pre-2000 surveys, causing our maps of transmission foci to reflect historical transmission patterns. Namibia has been experiencing dramatic declines in malaria burden over the past decade [27] and therefore no longer meets the steady state assumption required. While data were not available to confirm that malaria burden was stable pre-2000, overall burden during 2000–2004 was stable relative to the following decade, as active control efforts were limited [1,27]. Though our results therefore do not represent current transmission foci, they are a useful case study for areas considering pursuing elimination, and also allow us to examine changes in focal extent over time in Namibia by comparing with more recent burden data.

Our method predicts that several areas that appeared to sustain endemic transmission during the pre-elimination phase actually had unsustainable levels of transmission (Figs 4 and 5). This highlights the importance of accounting for movement, as this suggests parasite populations were maintained in some areas by non-endemic transmission alone, while similar steady-state analyses using classical models must conclude that these areas have local *R*_0_ > 1. The transmission landscape is also more heterogeneous when taking into account human mobility (Figs 4 and 5), agreeing with recent research that malaria is highly dynamic across space and time [4,12,39]. Areas where *R*_0_ > 1 are potentially important targets for malaria elimination planning in stable pre-elimination countries, as reducing focal transmission to locally unsustainable levels will cause the parasite population to propagate at below-replacement levels system wide [20].

Because this case study generates historical transmission foci maps, we compared the predicted spatial patterns of pre-elimination foci with 2009 incidence to determine how transmission patterns may have changed over time. Fig 6 juxtaposes incidence in 2009 from [26] with pre-elimination transmission foci. Districts overlapping foci had higher incidence than those outside foci (12.5 vs 8.7), suggesting that transmission in 2009 was higher in pre-elimination focal areas (Fig 6). Overall, however, the spatial pattern of burden in 2009 differed with predicted pre-elimination foci, and there were numerous extra-focal areas where 2009 incidence was high, such as in eastern Namibia. High incidence in these extra-focal areas could be caused by changes in transmission capacity patterns driven by climatic changes or heterogeneous application of control effort. Perhaps highlighted by its presence near national borders, high extra-focal incidence could also be driven by international importation [22], causing these extra-focal areas to be importation hotspots. The possibility of extra-focal importation hotspots near-elimination agrees with recent research on cross-border parasite mobility, which suggests that parasite importation generates significant amounts of onward transmission in areas with very low malaria endemicity [23,24]. Future passively detected health facility-level case data could refine the relationship between transmission foci, case distribution, and international human movement, as malaria control programs are increasingly recording travel history information when reporting cases. Populations in importation hotspots would be expected to have disproportionately high rates of travel, and malaria infected individuals in these areas will be more likely to have traveled to areas with higher transmission rates.

Though we applied this method to pre-elimination Namibia, this framework may be useful elsewhere if relevant population movement data are available and if the steady state assumption is satisfied. The prevalence estimates used in our Namibia case study are available globally [1,10], and while mobile phone data are not always available to parameterize movement, short-term movement can also be estimated using data informing other typologies of movement, including travel history surveys and census data [11]. These datasets should originate from the same period and should be very recent to be most useful operationally. In our case study, prevalence and movement data originated from different years (pre-elimination vs. 2010). Though this discrepancy is not ideal, recent studies suggest movement patterns are generally stable and predictable across temporal scales, potentially suggestive of temporal regularity in movement patterns [40,41]. These studies also suggest that though these types of movement are fundamentally different from those captured by mobile phone data, they likely exhibit similar patterns [40]. On the other hand, accurate and current entomological and parasitological parameters are often difficult to obtain, and in this study, estimates for these parameters originated from various entomological studies. We also assumed human recovery rates were homogeneous across all patches, though this parameter varies in reality due to differences in treatment-seeking behavior and health system coverage [42]. Though these estimates likely do not accurately reflect current malaria dynamics in Namibia, we found that model results are robust to uncertainty in entomological and parasitological parameters (see Methods). Varying recovery rates regionally based on recent studies on treatment-seeking behavior [42] also suggests limited effect on focal extent, as only 7 patches (or 2% of the 401 total patches) changed relative to the focus-defining criterion *R*_*0*_ *= 1*.

Further research will be necessary to extend utility of these analyses across transmission settings, particularly for countries with rapidly changing burden, as the presented analysis applies only to countries with stable and low levels of disease burden. In these countries, this framework could help target elimination campaigns on focal areas of transmission if elimination efforts are not currently ongoing. In the presented case study, we assume the transmission setting reflected in the prevalence surface used represents a period of relative stability, though pre-2000 burden data would be needed to confirm [1]. Dynamic prevalence is likely the rule rather than the exception, however, as countries with active elimination campaigns are often experiencing similar declines in transmission. Transmission is highly seasonal in many malaria endemic countries (including Namibia), as well. We did not incorporate seasonality because the Malaria Atlas Project model provided an average annual prevalence, rather than season-specific estimates [10]. This framework therefore will not be directly applicable to much of the globe until future work relaxes the steady state assumption and can account for seasonally and annually dynamic prevalence.

This framework would also be applicable to more areas if extended to use other metrics of malaria burden, as prevalence surveys become rarer and more uncertain near-elimination due to needing impractically large sample sizes. In such situations, country programs rely more heavily on clinical incidence measured at facilities [43], which could be used as an alternative source of disease burden information. As these data are a spatially heterogeneous subset of actual disease burden, using these data to reflect overall burden must account for factors such as test positivity rates and health facility catchment sizes, similar to the model used to estimate incidence in 2009 [26,42]. Importantly, recent research has better-defined the relationship between clinical incidence and prevalence, a useful step towards using clinical incidence data in this framework [44].

Despite these challenges applying this framework in near-elimination settings, it represents an important step towards understanding and mapping transmission foci on dynamic landscapes. The spatiotemporal dynamics of malaria are critical for elimination efforts, as transmission is known to exhibit spatial and temporal heterogeneity across multiple scales, and humans transport parasites between areas [20,21]. Prioritizing parasite sources requires a targeting algorithm that incorporates human movement and transmission heterogeneity [4]. This method accounts for these factors to produce maps of vectorial capacity and *R*_*0*_ that are operationally useful for targeting malaria control and elimination efforts. Outputs from this modeling framework can improve the outcomes of malaria control efforts and set a foundation for future targeting efforts across the spectrum of transmission for regional malaria elimination.

## Methods

### Ethics statement

This project was approved by Ethics and Research Governance of the University of Southampton (submission #7696).

### Malaria model

A basic version of the Ross-Macdonald model [45] has recently been updated to describe the spatial dynamics of malaria in a metapopulation [20]. The homogeneous version of this model forms the basis for the classical model without human mobility used in this manuscript:

$$\frac{dX}{dt}=mabY\left(1-X\right)-rX$$ (1)

$$\frac{dY}{dt}=acX\left(e^{-\mu \tau }-Y\right)-\mu Y$$ (2)

Here *X* and *Y* are the proportions of infected humans and vectors respectively. The parameters *r* and *μ* respectively represent the recovery rate of infected humans, and the death rate of infectious mosquitoes. The parameter *m* is the ratio of the total number of mosquitoes divided by the total number of humans, and both values are assumed constant through time. The parameter *a* is the rate at which mosquitoes bite humans, and *b* and *c* are the probabilities of a successfully disease-transmitting bite by an infectious mosquito on a susceptible human, and by an infectious human to a susceptible mosquito, respectively. The parameter *τ* is the incubation period in mosquitoes.

The behavior of this model is well-understood, and essentially depends on the value of the basic reproductive number *R*_*0*_, which relates the number of secondary cases expected from a single case in a completely naïve population:

$$R_{0}=\frac{ma^{2}bce^{-\mu \tau }}{r\mu }$$ (3)

Note that *R*_*0*_ can be factored as follows:

$$R_{0}=\left(\frac{ab}{\mu }\right)\left(\frac{mae^{-\mu \tau }}{r}\right)$$ (4)

From this factorization, we can interpret *R*_*0*_ as the product of the expected number of humans infected by a single infectious mosquito over its lifetime, and the expected number of infectious mosquitoes that arise from a single infectious human over their infectious period. If *R*_*0*_ < 1, then all solutions converge to the zero steady state, and malaria parasites are cleared from both mosquitoes and humans. If *R*_*0*_ > 1, then there is a unique stable endemic steady state to which all non-zero solutions converge.

A related malaria transmission measure is vectorial capacity, introduced in [25] as:

$$C=\frac{ma^{2}e^{-\mu \tau }}{\mu }=\left(\frac{a}{\mu }\right)\left(mae^{-\mu \tau }\right)$$ (5)

From the factorization, we can interpret *C* as the maximal possible rate at which a single infectious mosquito generates secondary infectious mosquitoes. *R*_*0*_ and *C* are related via the formula:

$$R_{0}=\frac{bc}{r}C$$ (6)

The multipatch version of this model is an *n* patch model in which a Ross-Macdonald model of the form Eqs (1) and (2) characterizes each patch, but various patches have different model parameters, indicated by subscripts *i = 1*, *…*, *n*. Each isolated patch model has its corresponding local basic reproduction number *R*_*0*,*i*_ and local vectorial capacity *C*_*i*_, and these quantities are defined as in Eqs (3) and (5). The multipatch model incorporates human movement by including the proportion of nights *p*_*i*,*j*_ that human residents of patch *i* spend in each patch *j* (where *p*_*i*,*i*_ is the proportion of nights residents of *i* spend at home). This movement model more closely resembles short-term movements away from the home than permanent or long-term movements [20]. These short-term movements are of increasing interest for understanding malaria, as significant importation of malaria can occur due to residents visiting areas with malaria, or through visitors from malaria endemic areas [22–24]. Only humans move in this model, both for simplicity and because we applied the model at larger spatial scales (*e*.*g*. patches larger than 50 km^2^) where vector movement is comparatively less important [11]. Since humans move, the reservoir of infectious humans that mosquitoes bite is not limited to a patch’s residents. We refer to this effective reservoir of infectious humans in patch *i* as *κ*_*i*_. *κ*_*i*_ can be calculated as a weighted sum of *X*_*j*_’s, or the proportion of infectious visitors from *j*, weighted by the proportion of time they spend in patch *i* (i.e. *p*_*j*,*i*_) and the sizes of the different patches, *H*_*j*_. *κ*_*i*_ is then given by the expression

$$\kappa_{i}=\frac{\sum_{j}p_{ji}X_{j}H_{j}}{\sum_{j}p_{ji}H_{j}}$$ (7)

Incorporation of these spatially heterogeneous effects yields the model:

$$\frac{dX_{i}}{dt}=\sum_{j}p_{i,j}m_{j}a_{j}b_{j}Y_{j}\left(1-X_{i}\right)-rX_{i}$$ (8)

$$\frac{dY_{i}}{dt}=a_{i}c_{i}\kappa_{i}\left(e^{-\mu_{i}\tau_{i}}-Y_{i}\right)-\mu_{i}Y_{i}$$ (9)

The proportion of infectious mosquitoes at any time is related to the proportion of the effective local human population that is infectious *κ*_*i*_. We solve eq (9) for the quasi-equilibrium proportion of infectious mosquitoes as before:

$$Y_{i}=\frac{c_{i}a_{i}\kappa_{i}}{\mu_{i}+c_{i}a_{i}\kappa_{i}}e^{-\mu_{i}\tau_{i}}$$

and substitute this expression into eq (8). This leads to the dynamics that describe the local transmission process in patch *i*:

$$\frac{dX_{i}}{dt}=\sum_{j=1}^{N}\frac{p_{ij}m_{j}a_{j}{}^{2}b_{j}c_{j}e^{-\mu_{j}\tau_{j}}\kappa_{j}}{a_{j}c_{j}\kappa_{j}+\mu_{j}}\left(1-X_{i}\right)-r_{i}X_{i}$$ (10)

### Calculating local vectorial capacities and local reproduction numbers with human mobility

Using the multipatch model, we estimate local transmission (*i*.*e*. *C*_*i*_ and *R*_*0*,*i*_) in a metapopulation that is assumed to be closed to immigration from outside the defined set of patches, and is assumed to be at its steady state with endemic malaria. In principle, the local transmission measures. *C*_*i*_ and *R*_*0*,*i*_ could be calculated from their respective definitions Eqs (3) and (5), but this requires that various model parameters in the patches are known. Here we will show how they can be determined from steady state measurements of the multipatch model, and certain patch parameter combinations.

The positive steady state of eq (10) can be written as:

$$\sum_{j=1}^{N}p_{ij}b_{j}c_{j}\frac{k_{j}}{\frac{a_{j}c_{j}k_{j}}{\mu_{j}}+1}C_{j}=\frac{r_{i}X_{i}}{1-X_{i}},\;i=1,\ldots ,n$$ (11)

or in matrix form as *AC* = *g*(*X*), where

$$g\left(X\right)=\left(\begin{array}{c} g_{1}\left(X\right)\\ \vdots \\ g_{n}\left(X\right) \end{array}\right)\text{with},g_{i}\left(X\right)=\frac{r_{i}X_{i}}{1-X_{i}},\;C=\left(\begin{array}{c} C_{1}\\ \vdots \\ C_{n} \end{array}\right),$$ (12)

and

A=Pdiag(f(X)), (13)

where *P* is the connectivity matrix having *p*_*ij*_ as its (*ij*)th entry, and where

$$f\left(X\right)=\left(\begin{array}{c} f_{1}\left(X\right)\\ \vdots \\ f_{n}\left(X\right) \end{array}\right)\;f_{i}\left(X\right)=b_{i}c_{i}\frac{\kappa_{i}}{\frac{a_{i}c_{i}}{\mu_{i}}\kappa_{i}+1}.$$ (14)

Therefore, if *A* is invertible, we can calculate the vector of local transmission capacities:

$$C=A^{-1}g\left(X\right)$$ (15)

Note that to calculate *C* with this approach, we need the values of the following parameters, and parameter combinations:

1. The steady state vector *X* of infectious proportions of people in the various patches.
2. The connectivity matrix *P*.
3. The recovery rates *r*_*i*_ in each patch (to evaluate *g*_*i*_*(X)*).
4. The product *b*_*i*_*c*_*i*_ for each patch. This term is the product of the probabilities of infectious bites from vector to host and host to vector.
5. The parameter combination *a*_*i*_*c*_*i*_*/μ*_*i*_ in each patch. This represents the total number of possible times a mosquito in patch *i* could get infected, assuming it only bites infected humans.
6. The total number of humans *H*_*i*_ in each patch (to evaluate *κ*_*i*_, see Eq (5)).

After calculating all values of *C*_*i*_, the corresponding values of *R*_*0*,*i*_ can be determined from the relationship *R*_0_,_*i*_ = (*b*_*i*_*c*_*i*_/*r*_*i*_)*C*_*i*_. We note that no additional parameters are needed in this case since *b*_*i*_*c*_*i*_ and *r*_*i*_ were needed in the calculation of the vector of local transmission capacities.

Alternatively, the local reproduction numbers *R*_*0*,*i*_ can be calculated directly by reformulating the steady state expression as:

$$\sum_{j=1}^{N}p_{ij}r_{j}\frac{k_{j}}{\frac{a_{j}c_{j}k_{j}}{\mu_{j}}+1}R_{0,j}=\frac{r_{i}X_{i}}{1-X_{i}},\;i=1,\;\ldots ,\;n,$$ (16)

and a similar matrix inversion leads to the desired result. In this case, we need the values of *X*_*i*_, *p*_*ij*_, *r*_*i*_, *a*_*i*_*c*_*i*_*/μ*_*i*_, and *H*_*i*_, which are the same as in the calculation of the local transmission capacities, except *b*_*i*_*c*_*i*_ is no longer needed. If we make the assumption that the recovery rates *r*_*i*_ in all patches are equal, then they cancel out in the above equations, and are no longer needed to calculate local reproduction numbers.

Testing reasonable ranges for the necessary entomological parameters *a*, *μ*_*i*_, and *c* (0.03–1, 0.1–0.9, and 0.1–0.9, respectively), we found that while absolute values of *R*_*0*_ estimates changed slightly, no patches changed relative to the focus-defining criterion *R*_*0*_ *= 1*. We tested the effect of using recovery rates adjusted by probabilities of seeking treatment from [26], aggregated to region, and applied to all patches within a particular region. We made the conservative estimate that if an individual sought treatment for malaria, they were only infectious for one day, while individuals that didn’t seek treatment took the entire period to recover. The effective recovery rate in a given patch was then $\frac{r\left(1-t\right)+t}{2}$, where *t* is the proportion of people who sought treatment when feverish. While most patches changed slightly in absolute *R*_*0*_ estimate, only 7 changed relative to the criterion *R*_*0*_ *= 1* under these conditions. We also assigned recovery rates randomly to patches bounded by the estimates provided by [42] (bounding *t* by the region-level minimum and maximum estimates of 27.1% and 58.3%) to represent an extreme case of heterogeneity in treatment-seeking behavior. Across 1000 random assignments the number of patches that changed relative to *R*_*0*_ *= 1* was at most 45, or 11% of all patches.

### Pre-elimination prevalence estimates

To quantify *R*_*0*,*i*_ using prevalence estimates, or estimates reflecting the proportion of people infected with malaria (*X*_*i*_ in our model), we used a gridded prevalence surface estimated from parasite rate surveys [10]. This gridded surface is freely available at http://www.map.ox.ac.uk/. This continuous parasite rate surface was created using a collection of historical *Plasmodium falciparum* parasite rate (*Pf*PR) surveys and remotely sensed across the globe such that surveys near a given point were weighted by distance to inform prevalence at that point. This predicted surface was validated by comparing predicted with observed prevalence in randomly selected surveys and by comparing classification accuracy based on low, intermediate, and high endemicity classes (defined as <5%, 5–40%, and >40% *PfPR*, respectively). Validation statistics were calculated using a hold-out subset of the surveys with a model specifically fitted without these held-out data. Overall, there was a global mean error of only -0.56% and 79.5% of surveys were classified as the correct endemicity class, suggesting that this surface is generally accurately predicting parasite rate.

In Namibia, the prevalence surveys used originated from 1985 and 1990, while data for neighboring Botswana included 1997 surveys and Angola included 2006 and 2007 surveys. Because most of the data that would inform prevalence across Namibia then originate from before 2000, we assume that this surface represents a picture of prevalence throughout Namibia before elimination efforts were scaled up in 2004.

Each patch was a Voronoi polygon around a settlement centroid defined as a combination of urbanized areas, yielding 402 total patches. The population within each patch *H*_*i*_ was calculated using WorldPop population estimates from 2010 (freely available from http://www.worldpop.org). We then defined prevalence in each patch as the mean PR for each polygon.

### 2009 incidence estimates

In this study, we compare the predicted map of transmission foci with more recent incidence estimates from [26], which are presented in this manuscript as-is. These incidence estimates were modeled at the constituency level (second level administrative unit; 108 total) and modeled incidence using routinely collected health management information systems (HMIS) data. Because these estimates only reflect cases that presented at health facilities, this incidence map used a model of treatment seeking behavior to adjust observed malaria cases based on test positivity rates and health facility utilization and define health facility catchment populations. The catchment population model was calibrated using a malaria indicator survey from 2009 in Namibia. The final model was the best of several models tested compared using deviance information criterion and the conditional predictive ordinate. The authors tested overall predictive performance of the final model by calculating a Pearson correlation coefficient for the best model using a hold-out set, which was calculated to be 0.56. These incidence estimates represent a highly transient near-elimination period, as malaria burden has declined steadily since 2004 [27].

### Namibia anonymized mobile phone call data records (CDRs)

We pair the pre-elimination prevalence surface with individual-level movement patterns obtained from a mobile phone dataset from Namibia, originating from 2010 to map historical transmission foci. Though the time period of the movement and prevalence datasets, recent studies suggest that human movement is typically regular and predictable across temporal scales [40] and recently-acquired mobile phone data from Namibia (2011–2014) suggest that movement patterns have been broadly regular seasonally and stable across years. Because of this, and because of Namibia’s political stability since independence in 1990, we assume that these 2010 mobile phone data likely reflect movement patterns similar to those that would have been observed in 2000.

From October 2010 to September 2011, a total of 9 billion communications from 1.19 million unique SIM cards were identified in the dataset, representing 85% of the estimated 1.4 million adult (aged over 15 years old) population of Namibia United Nations, “Volume I: Comprehensive Tables.”. We obtained these data through written agreements between Mobile Telecommunications Limited (MTC), the NVDCP, and the Clinton Health Access Initiative (CHAI). These data are owned by MTC, who provided permission for publication of this manuscript given its use of these data. A map of cell tower coverage can be found online at http://www.mtc.com.na/coverage, and S1 Fig shows population density estimates throughout Namibia obtained from the WorldPop project for comparison of population densities in areas covered by cell towers.

In this dataset, each of the call data records was associated with one of 402 settlements across Namibia (representing the origin tower for each call), corresponding to settlement centroids defined as a combination of urbanized areas. Using this data, we collected all the records corresponding to each SIM. When people made multiple calls or texts in a given day, we only used the last CDR event of the day, assuming that this represented the best estimate of where they spent the night. We therefore only included movement that resulted in overnight stays in a different location, as malaria vectors in Namibia generally bite at night [27]. When sequential CDR events occurred at different locations, we assumed the person moved from the location of the initial CDR to the location of the subsequent CDR at the time halfway between the two CDRs. In this way, we were able to estimate the location of individual SIMs over the one year period, and therefore determine the proportion of nights spent in service of each cell phone tower (each tower corresponding to a patch in our analysis).

**Fig 7:**
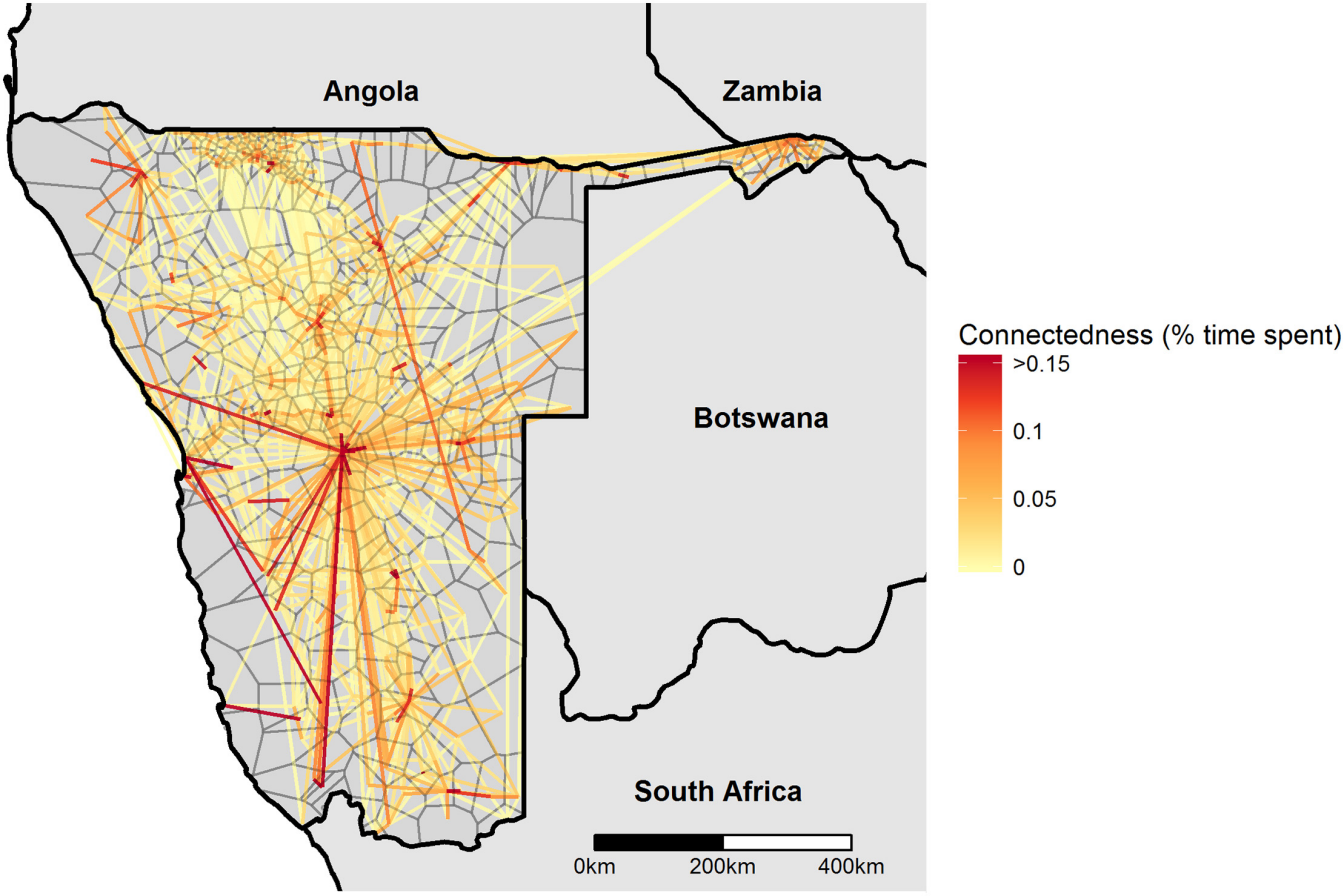
Patch-level connectivity. Patch boundaries drawn using Voronoi polygons around cell towers shown in thin black lines. Connectivity shown between patch centroid, with line color (yellow/red) representing the proportion of time people in each patch spend in the other (average of both directions shown). Higher values indicate better-connected pairs of patches. Only the top 2% of pairwise connections are shown.

For each SIM, we assumed that an individual’s home patch was the patch where an individual spent most of their time. The connectivity matrix was defined by calculating *p*_*ij*_ as the mean amount of time residents of patch *i* spent in *j*. Fig 7 shows the connectivity of different patches in the CDR dataset. A version of this dataset aggregated to the constituency level can be found in the Supplementary Information (S1 Table; constituencies corresponding to those found in the administrative unit 2 shapefile provided for Namibia by www.gadm.org). Several constituencies did not contain any cell towers, so are not connected to any others in this aggregated dataset.

## Supporting Information

S1 Fig Population density with Voronoi polygons around cell towers. Thick gray lines indicate national borders, while thin gray lines within Namibia represent Voronoi polygon (used as patches in our model) borders. Each pixel in the population raster is a 10 x 10km grid square.
(TIF)

S1 Table Adjacency matrix from mobile phone data aggregated to the constituency level. Element [i,j] refers to the proportion of time residents in constituency i spent in constituency j. Rows that sum to zero represent constituencies that had no cell towers within them.
(CSV)
