# Supplementary figures and images for: Identifying Malaria Transmission Foci for Elimination Using Human Mobility Data
Source: PLoS Comput Biol. 2016 Apr 4;12(4):e1004846. doi: 10.1371/journal.pcbi.1004846 (PMC4820264; doi:10.1371/journal.pcbi.1004846)

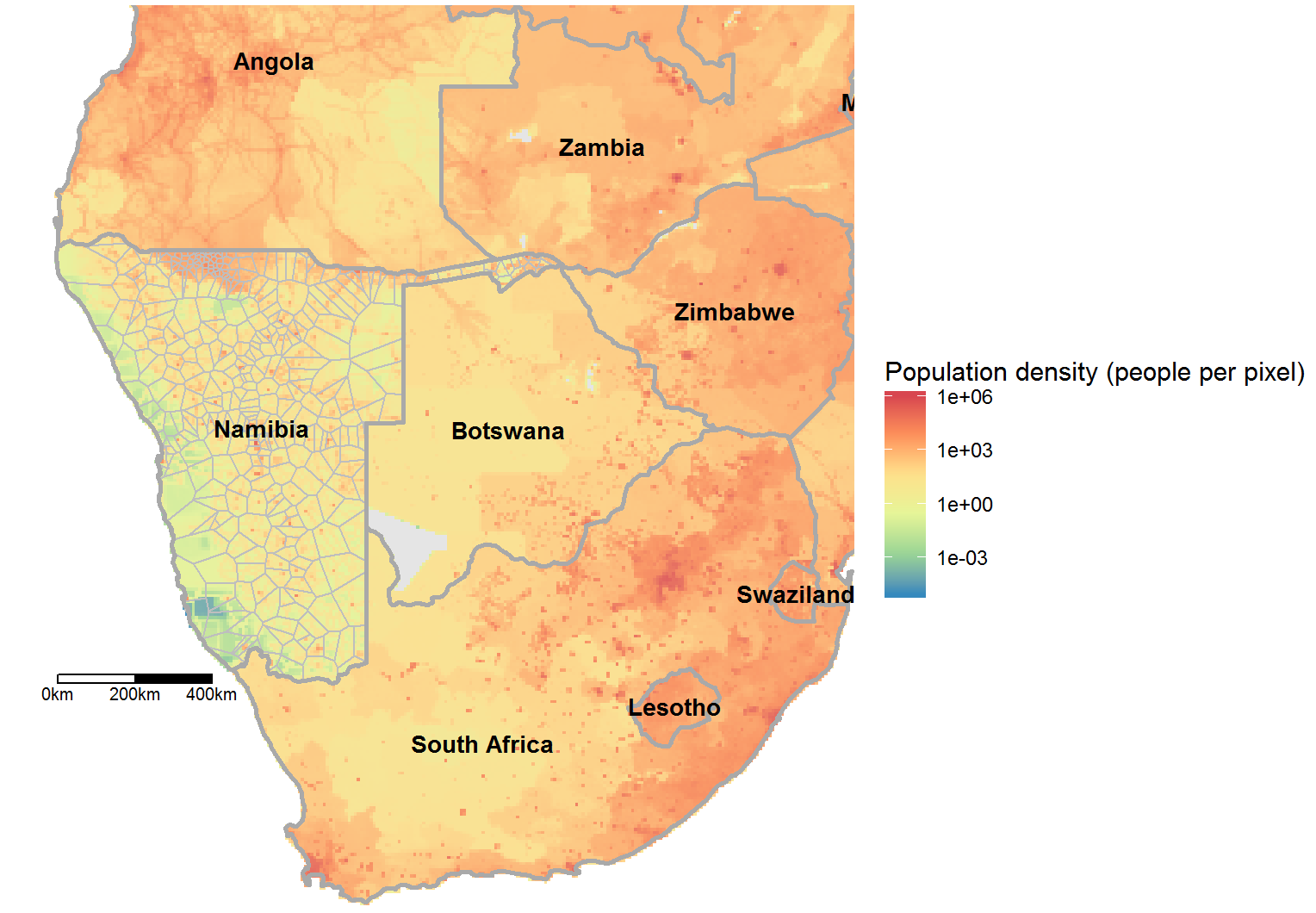

Supplement: S1 Fig — Thick gray lines indicate national borders, while thin gray lines within Namibia represent Voronoi polygon (used as patches in our model) borders. Each pixel in the population raster is a 10 x 10km grid square. (TIF) [file pcbi.1004846.s001.tif]
